# Habitual Constipation

**Published:** 1888-05

**Authors:** 


Habitual Constipation.—W. J. Maddox, M. D., Washington, D. C., says: In regard to results produced from the use of Acid Mannate, I will give two cases: Case 1, Mrs. N. C., applied to me for treatment for habitual constipation. After trying several remedies without any good effect, I put her on the Acid Mannate treatment. Since taking it, she has had marked improvement, and at this date is not troubled with constipation. Case 2, Mrs. F., pregnant, was troubled with constipation. I gave the Acid Mannate, and find that it acted like a charm with her. She, at the present time, is not constipated. Both of the above patients told me that the Acid Mannate operated very mildly. It is the remedy for constipation, either habitual or caused from pregnancy. I shall continue to use it, being very much pleased with its action.

